# Factors Associated With Extrapulmonary Tuberculosis in Southeastern Mexico: A Case-Control Study

**DOI:** 10.7759/cureus.59739

**Published:** 2024-05-06

**Authors:** Jorge Fernando Luna-Hernández, María Del Pilar Ramírez-Díaz, Abel Eduardo Zavala, Doireyner Daniel Velázquez-Ramírez, Gabriel Hernández-Ramírez, Edna Isabel Rodríguez-López

**Affiliations:** 1 Biological and Health Sciences, University of the Isthmus, Juchitán, MEX; 2 Health and Welfare, Inter-American Conference on Social Security, Ciudad de México, MEX; 3 Emerging and Epidemic Diseases, The South Border College (ECOSUR), San Cristobal de las Casas, MEX

**Keywords:** mycobacterium infections, bacterial infections, population of risk, risk factors, extrapulmonary tuberculosis, mycobacterium tuberculosis

## Abstract

Introduction: Extrapulmonary tuberculosis (EPTB) is a disease that can affect any organ or tissue. Due to its potential to cause more dangerous sequelae and the barriers to its timely diagnosis, greater clinical awareness of this disease is crucial. This study aimed to identify the factors associated with EPTB in the population of Oaxaca, Mexico.

Methods: This is an unpaired case-control study. The cases were patients with EPTB+ while the controls were patients with pulmonary tuberculosis (PTB+) registered in the Tuberculosis Epidemiological Surveillance System. Sociodemographic, clinical, and microbiological variables were recovered. Bivariate analyses were performed and logistic regression analyses were performed to calculate the odds ratio (OR).

Results: A total of 75 EPTB+ cases and 300 PTB+ controls were included. Of the total sample, 57.1% were men and 60.3% indigenous. The most frequent clinical presentations of EPTB+ were nodal (21.3%), miliary (21.3%), and breast (20.0%). According to logistic regression analysis, age <40 years (OR: 2.25 (95% CI: 1.13-4.49), female sex (OR: 1.92 (95% CI: 1.03-3.56)], urban residence (OR: 2.25 (95% CI: 1.11-4.55)), comorbidity with human immunodeficiency virus/acquired immunodeficiency syndrome (HIV/AIDS) (OR: 3.46 (95% CI: 1.31-9.10)), dyspnea (OR: 2.67 (1.22-5.82)), and adenopathy (OR: 3.38 (95% CI: 1.42-8.06)) were positively associated with EPTB+.

Conclusion: These results can serve as a basis for screening EPTB+, thus improving the preventive and diagnostic capacity of local health services, taking as a starting point women under 40 years of age and patients with HIV/AIDS in urban areas, as well as the presence of adenopathy and dyspnea as clinical characteristics of the disease.

## Introduction

Tuberculosis (TB) is a communicable disease that remains as one of the leading causes of death worldwide. Before the COVID-19 pandemic, TB was the leading cause of death by a single infectious agent [1]. It is estimated that a quarter of the world's population has been infected with TB; however, most of these people will not develop active disease, with approximately 10 million active cases reported annually [1,2]. On the other hand, global mortality has reflected an increase from 1.5 million in 2020 to 1.6 million deaths in 2021, which represents a setback in the goals stipulated for TB elimination [1].

Pulmonary tuberculosis (PTB) is the most frequent clinical presentation; however, the bacillus can affect other parts of the body, which is called extrapulmonary tuberculosis (EPTB). In 2019, EPTB accounted for 16% of the 7.1 million TB cases reported worldwide, while, in Mexico, the proportion of EPTB has been estimated at 20% of the total TB cases reported [3,4]. The pathogenesis of EPTB usually consists of a primary lung disease with hematogenous and lymphatic dissemination of the bacillus to other parts of the body; however, this can occur without lung infection [5]. It affects any organ or tissue except hair, nails, and teeth, with lymph nodes, pleura, skin tissue, abdomen, gastrointestinal system, and bones being the most affected sites [6-9].

EPTB has been less studied, perhaps because of its less contagious characteristics and, therefore, its low contribution to the spread of TB [10]. However, this modality can spread to the lungs and become contagious, posing a latent threat to public health [10]. Furthermore, because of its potential to result in higher hazard sequelae such as those related to the nervous system and the barriers to its timely diagnosis, particularly in immunocompromised individuals and vulnerable groups such as people with diabetes, better clinical knowledge about EPTB is crucial [7,11-13]. The proportion and clinical occurrence of EPTB varies with geographical, social, ethnic, biological, and economic parameters [8]. However, in Mexico, they have been studied less [14]. From this perspective, the objective of this study was to identify the factors associated with EPTB compared to PTB in the population of Oaxaca, Mexico.

## Materials and methods

We conducted an unpaired case-control study on the population living in the Isthmus of Tehuantepec, located in Oaxaca, Mexico. This region is one of the eight regions comprising the state of Oaxaca and has 629,036 inhabitants, of which 60% live in poverty and 61% claim to be indigenous.

Recovery of study subjects and variables

**Figure 1:**
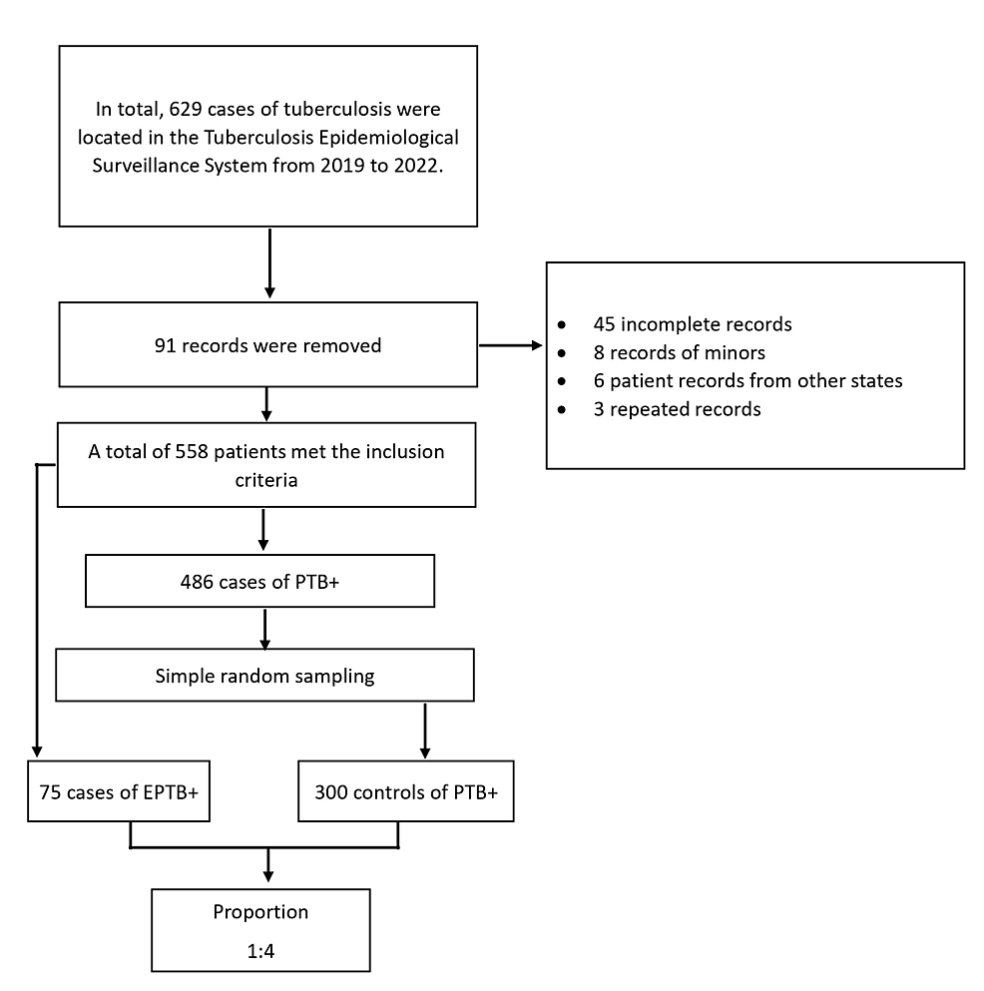
Selection process for the study cases and controls Selection process for cases and controls from clinical records registered in the Tuberculosis Epidemiological Surveillance System, which is part of Health Jurisdiction No. 2 located in the Isthmus of Tehuantepec, Oaxaca, Mexico.

The inclusion criteria for the cases were subjects clinically and microbiologically diagnosed with EPTB+ and who did not have convergent PTB. Controls were patients with PTB+ exclusively registered in the Tuberculosis Epidemiological Surveillance System of the Health Jurisdiction No. 2, Isthmus Region. Minors were excluded, and also repeated and incomplete records were eliminated. All cases of EPTB+ that met the inclusion criteria were selected. The selection of PTB+ controls was done by simple random sampling, leaving a ratio of one case to four controls. Records from 2019 to 2022 were included (Figure 1).

Sociodemographic variables were retrieved from medical records such as age, sex, occupation, education level, indigeneity, socioeconomic level (SES), and type of population (rural: <2500 inhabitants or urban: ≥2500 inhabitants); clinical variables such as body mass index (BMI); comorbidities such as type 2 diabetes mellitus (DM2), human immunodeficiency virus/acquired immunodeficiency syndrome (HIV/AIDS), neoplasms, symptomatology, alcohol, tobacco and drug use; and microbiological characteristics variables included type of case, type of diagnosis, diagnostic outcome, presence of drug resistance, previous contact with subjects infected with TB, treatment regimen, and antecedent of BCG (bacillus Calmette-Guérin) vaccination.

Data processing and statistical analysis

Data were processed in the SPSS Statistics for Windows, Version 27 (Armonk, NY: IBM Corp). Qualitative variables were presented as frequencies and proportions, and quantitative variables as means, medians, standard deviations, and interquartile ranges (IQR).

For proportional differences between qualitative variables, the Chi-squared test or Fisher's Exact test was used depending on the number of observations per cell, while for quantitative variables Mann-Whitney U or Student's T-test was used according to data distribution. Bivariate analyses were performed. To identify the associated factors, a binary logistic regression was used to calculate the odds ratios (ORs) with their 95% confidence intervals. For adjustment, variables that were significant in the bivariate analysis or reported as risk factors for EPTB+ were taken into account. The Hosmer and Lemeshow test for goodness of fit was considered (p>0.05).

Ethical considerations

This study was carried out in accordance with the Declaration of Helsinki. It is a low-risk study according to the General Health Law since secondary information was used, which does not imply any risk to the health of the study subjects. We had the consent of the corresponding authorities of Health Jurisdiction No. 2, who provided the information under the principle of confidentiality.

## Results

A total of 375 participants were included, of whom 75 (20%) were EPTB+ and 300 (80%) were PTB+ with a median age of 51 years (IQR: 38-64).

Demographic variables

Of the total sample, 214 (57.1%) were men and 226 (60.3%) reported being indigenous. More than 238 (60%) had a low level of education, and just over 118 (31.5%) were engaged in the household, 99 (26%) in primary activities and 84 (22.4%) were employees or teachers. Of the population, 238 (63.5%) had a low SES, and 248 (66%) were residents of an urban population. The median age of patients with EPTB+ was 37 years (IQR: 28-59) and of participants with PTB+ was 53 (IQR: 43-65) showing significant differences (p=<0.001). Also, a higher proportion of patients with EPTB+ residing in urban areas (p=0.005) and with a higher SES (p=<0.001) was observed (Table 1).

**Table 1:** Sociodemographic characteristics of the study population ^a^Chi-squared test ^b^Mann-Whitney U Test ^c^Fisher's exact test ^ѧ^Different symbols indicate percentage differences EPTB, extrapulmonary tuberculosis; PTB, pulmonary tuberculosis; IQR, interquartile range; SES, socioeconomic status

	Total	EPTB+	PTB+	
	n=375 (%)	n=75 (%)	n=300 (%)	p
Age				
Median (IQR)	51 (38-64)	37 (28-59)	53 (43-65)	0.007^b^
Sex				
Male	214 (57.1)	31 (41.3)	183 (61.0)	0.002
Female	161 (42.9)	44 (58.7)	117 (39.0)	
Indigenism				
Yes	226 (60.3)	30 (40.0)	196 (65.3)	<0.001
No	149 (39.7)	45 (60.0)	104 (34.7)	
Level of education				
No formal education	88 (23.5)	8 (10.7)	80 (26.7)	
Elementary	150 (40.0)	24 (32.0)	126 (42.0)	<0.001
Secondary/high school	88 (23.5)	30 (40.0)	58 (19.3)	
Professional/postgraduate	49 (13.1)	13 (17.3)	36 (12.0)	
Occupation				
Unemployed	28 (7.5)	8 (10.7)	20 (6.7)	
Home	118 (31.5)	24 (32.0)	94 (31.3)	
Workers/drivers/professions	24 (6.4)	2 (2.7)	22 (7.3)	
Primary activities	99 (26.4)	15 (20.0)	84 (28.0)	
Employed/teachers/others	84 (22.4)	18 (24.0)	66 (22.0)	0.080^c^
Students	4 (1.1)	3 (4.0)	1 (0.3)	
Health workers	6 (1.6)	3 (4.0)	3 (1.0)	
Retired/pensioner	11 (2.9)	2 (2.7)	9 (3.0)	
Deprived of liberty	1 (0.3)	0 (0.0)	1 (0.3)	
Socioeconomic status				
Low	238 (63.5)	13 (17.3)^ѧ^	36 (12.0)^ѧ^	
Medium	88 (23.5)	30 (40.0)^ѧ^	58 (19.3)^ѥ^	<0.001
High	49 (13.1)	32 (42.7)^ѧ^	206 (68.7)^ѥ^	
Residence				
Rural	127 (33.9)	15 (20.0)	112 (37.3)	0.005
Urban	248 (66.1)	60 (80.0)	188 (62.7)	

Clinical variables

It was observed that 66 (17.6%) were overweight (SP) and 38 (10.1%) were obese (OB). In addition, 97 (25.9%) presented comorbidity with DM2 and 18 (4.8%) with HIV. The population studied reflected a low consumption of alcohol, tobacco, and drugs. On the other hand, the signs and symptoms with the highest proportion were productive coughs in 271 patients (72.3%), weight loss in 303 (80.8%), and muscle weakness in 256 (68.3%). As for the comparison between EPTB+ and PTB+, dyspnea, adenopathy, and comorbidity with HIV/AIDS were more frequent in patients with EPTB+ (p=0.008), (p=<0.001), and (p=0.008), respectively. The most frequent clinical presentations of EPTB+ were lymph node in 16 patients (21.3%), miliary in 16 (21.3%), and breast in 15 (20.0%) patients (Table 2).

**Table 2:** Clinical characteristics of the study population ^a^Chi-squared test ^b^Fisher's exact test N/A, no apply; BMI, body mass index; CSN, central nervous system; EPTB, extrapulmonary tuberculosis; PTB, pulmonary tuberculosis; HIV/AIDS, human immunodeficiency virus/acquired immunodeficiency syndrome

	Total	EPTB+		PTB+			
	n=375		n=75		n=300		P^a^
BMI classification							
Underweight	6 (1.6)		10 (13.3)		50 (16.7)		
Normal	211 (56.3)		39 (52.0)		172 (57.3)		0.492
Overweight	66 (17.6)		17 (22.7)		49 (16.3)		
Obesity	38 (10.1)		9 (12.0)		29 (9.7)		
Comorbidities							
HIV/AIDS	18 (4.8)		8 (10.7)		10 (3.3)		0.008
Diabetes	97 (25.9)		8 (10.7)		89 (29.7)		<0.001
Neoplasms	5 (1.3)		0 (0.0)		5 (1.7)		0.260^b^
Lifestyle variables							
Alcohol use	42 (11.2)		5 (6.7)		37 (12.3)		0.164
Tobacco use	20 (5.3)		1 (1.3)		19 (6.3)		0.085
Drug use	10 (2.7)		1 (1.3)		9 (3.0)		0.694^b^
Clinical manifestations							
Dyspnea	50 (13.3)		17 (22.7)		33 (11.0)		0.008
Adenopathy	38 (10.1)		19 (25.3)		19 (6.3)		<0.001
Productive cough	271 (72.3)		23 (30.7)		248 (82.7)		<0.001
Fever	254 (67.7)		36 (48.0)		218 (72.7)		<0.001
Asthenia	227 (60.5)		32 (42.0)		195 (65.0)		<0.001
Adynamia	216 (57.6)		34 (45.3)		182 (60.7)		0.016
Diaphoresis	154 (41.1)		21 (28.0)		133 (44.3)		0.010
Headache	183 (48.8)		27 (36.0)		156 (52.0)		0.013
Weakness	256 (68.3)		44 (58.7)		212 (70.7)		0.046
Hemoptysis	77 (20.5)		4 (5.3)		73 (24.3)		<0.001
Weight loss	303 (80.8)		53 (70.7)		250 (83.3)		0.013
Muscular weakness	179 (47.7)		24 (32.0)		155 (51.7)		0.002
Type of EPTB							
Ganglionic	16 (4.3)		16 (21.3)		N/A		N/A
Miliary	16 (4.3)		16 (21.3)		N/A		N/A
Breast	15 (4.0)		15 (20.0)		N/A		N/A
Pleural	9 (2.4)		9 (12.0)		N/A		N/A
Intestinal/peritoneal	7 (1.9)		7 (9.3)		N/A		N/A
Osteoarticular	6 (1.6)		6 (8.0)		N/A		N/A
Meningeal/CSN	2 (0.5)		2 (2.7)		N/A		N/A
Cutaneous	2 (0.5)		2 (2.7)		N/A		N/A
Renal	1 (0.3)		1 (1.3)		N/A		N/A
Rectal/anal	1 (0.3)		1 (1.3)		N/A		N/A

Characteristics of the type of case, diagnosis, and treatment

Regarding microbiological characteristics, 358 (95.5%) were new cases and diagnosed mainly by smear microscopy (210, 56.0%) and radiography (129, 34.4%). Only 248 (66%) had a history of BCG vaccination, six (1.6%) presented some type of antibiotic resistance, and 79 (21.1%) reported having had previous contact with someone with TB, with patients with PTB+ having the most contact (p=<0.001) (Table 3).

**Table 3:** Characteristics of the type of case, diagnosis, and treatment ^a^Chi-squared test ^b^Fisher's exact test ^c^Includes relapse to primary treatment and retreatment, readmissions due to abandonment, and failure to treatment or retreatment PCR, polymerase chain reaction; TB, tuberculosis; MTB, Mycobacterium tuberculosis; RIF, rifampicin; CAT, computed axial tomography; BAAR, acid-alcohol-resistant bacillus; BCG, bacillus Calmette-Guérin; MDR, multidrug-resistant; EPTB, extrapulmonary tuberculosis; PTB, pulmonary tuberculosis

	Total		EPTB+		PTB+					
	n=375	n=75				n=300	P^a^			
Case type										
New case	358 (95.5)	74 (98.7)				284 (94.7)	0.058			
Others^c^	17 (4.5)	1 (1.3)				16 (5.3)				
Diagnosis type										
Bacilloscopy	207 (55.2)	1 (1.3)				206 (68.7)				
Culture	8 (2.1)	4 (5.3)				4 (1.3)				
Histopathology	18 (4.8)	16 (21.3)				2 (0.7)				
PCR/Xpert MTBRIF	3 (0.8)	2 (2.7)				1 (0.3)	N/A			
Radiography	129 (34.4)	44 (58.7)				85 (28.3)				
CAT	7 (1.9)	5 (6.7)				2 (0.7)				
Urine test	3 (0.8)	3 (4.0)				0 (0.0)				
Diagnostic result										
(1 a 9 BAAR)	8 (2.1)	1 (1.3)				7 (2.3)				
(+)	69 (18.4)	2 (2.7)				67 (22.3)				
(++)	66 (17.6)	1 (1.3)				65 (21.7)				
(+++)	69 (18.5)	0 (0.0)				69 (23.0)	N/A			
Suggestive data for TB	155 (41.4)	65 (82.6)				90 (30.0)				
Positive (culture)	5 (1.3)	4 (5.3)				1 (0.3)				
MTB	3 (0.8)	2 (2.7)				1 (0.3)				
Treatment scheme										
Primary	353 (94.1)	71 (94.7)				282 (94.0)				
Shortened retreatment	5 (1.3)	1 (1.3)				4 (1.3)				
Standardized retreatment	2 (0.5)	0 (0.0)				2 (0.7)	N/A			
Individualized retreatment	1 (0.3)	0 (0.0)				1 (0.3)				
Did not start treatment	15 (3.8)	4 (3.3)				11 (3.7)				
BCG background	248 (66.1)	58 (77.3)				190 (63.3)	0.022			
Previous contact with TB patient	79 (21.1)	3 (4.0)				76 (25.3)	<0.001^b^			
Drug resistance										
Monoresistance	5 (1.3)	1 (1.3)				4 (1.3)				
MDR	1 (0.3)	0 (0.0)				1 (0.3)	0.882^b^			
No resistance	369 (98.4)	74 (98.7)				295 (98.3)				

Identification of risk factors

It was observed that being female, <40 years old, residents of urban population, higher SES, comorbidity with HIV, history of BCG vaccination, dyspnea, adenopathy, and comorbidity with HIV/AIDS were variables associated with EPTB+. While indigeneity, presence of DM2, and no previous contact with TB subjects decreased the probability of EPTB+ in the crude analysis (Table 4).

**Table 4:** Estimation of crude ORs for factors associated with EPTB+ ^a^Chi-squared test ^b^Fisher's exact test ^├^crude OR 1, reference category; OR, odds ratio; BMI, body mass index; PTB, pulmonary tuberculosis; HIV/AIDS, human immunodeficiency virus/acquired immunodeficiency syndrome; EPTB, extrapulmonary tuberculosis; TB, tuberculosis

	Total (%)	EPTB+ (%)	PTB+ (%)	OR^├^	CI 95%	P^a^
	n=375 (100)	n=75 (20)	n=300 (80)			
Age						
<40 years	105 (28.0)	41 (54.7)	64 (21.3)	4.44	(2.61-7.56)	<0.001
>40 years	270 (72.0)	34 (45.3)	236 (78.7)		1	
Sex						
Female	161 (42.9)	44 (58.7)	117 (39.0)	2.22	(1.32-3.71)	0.002
Male	214 (57.1)	31 (41.3)	183 (61.0)		1	
Indigenism						
Indigenous	226 (60.3)	30 (40.0)	196 (65.3)	0.35	(0.21-0.59)	<0.001
Non-indigenous	149 (39.7)	45 (60.0)	104 (34.7)		1	
Population						
Urban	248 (66.1)	60 (80.0)	188 (62.7)	2.38	(1.29-4.39)	0.005
Rural	127 (33.9)	15 (20.0)	112 (37.3)		1	
Socioeconomic status						
Medium/high	137 (36.5)	43 (57.3)	94 (31.3)	2.94	(1.75-4.94)	<0.001
Low	238 (63.5)	32 (42.7)	206 (68.7)		1	
HIV/AIDS						
Yes	18 (4.8)	8 (10.7)	10 (3.3)	3.46	(1.31-9.10)	0.008
No	357 (95.2)	67 (89.3)	290 (96.7)		1	
Diabetes						
Yes	97 (25.9)	8 (10.7)	89 (29.7)	0.28	(0.13-0.61)	0.001
No	278 (74.1)	67 (89.3)	211 (70.3)		1	
BMI						
>25 kg/m	104 (27.7)	26 (34.7)	78 (26.0)	1.51	(0.87-2.59)	0.134
<25 kg/m	271 (72.3)	49 (65.3)	222 (74.0)		1	
Alcohol use						
Yes	42 (11.2)	5 (6.7)	37 (12.3)	0.50	(0.19-1.34)	0.164
No	333 (88.8)	70 (93.3)	263 (87.7)		1	
Tobacco use						
Yes	20 (5.3)	1 (1.3)	19 (6.3)	0.20	(0.02-1.51)	0.064^b^
No	355 (94.7)	74 (98.7)	281 (93.7)		1	
Drugs use						
Yes	10 (2.7)	1 (1.3)	9 (3.0)	0.43	(0.54-3.50)	0.694
No	365 (97.3)	74 (98.7)	291 (97.0)		1	
BCG vaccine						
Yes	248 (66.1)	58 (77.3)	190 (63.3)	1.97	(1.09-3.56)	0.022
No	127 (33.9)	17 (22.7)	110 (36.7)		1	
Contact with TB						
Yes	79 (21.1)	3 (4.0)	76 (25.3)	0.12	(0.03-0.40)	<0.001
No	296 (78.9)	72 (96.0)	224 (74.7)		1	
Drug resistance						
Yes	6 (1.6)	1 (1.3)	5 (1.7)	0.79	(0.09-6.92)	0.837
No	369 (98.4)	74 (98.7)	295 (98.3)		1	
Adenopathy						
Yes	38 (10.1)	19 (25.3)	19 (6.3)	5.01	(2.49-10.08)	<0.001
No	337 (89.9)	56 (74.7)	281 (93.7)		1	
Dyspnea						
Yes	50 (13.3)	17 (22.7)	33 (11.0)	2.37	(1.23-4.54)	0.008
No	325 (86.7)	58 (77.3)	267 (89.0)		1	

**Figure 2:**
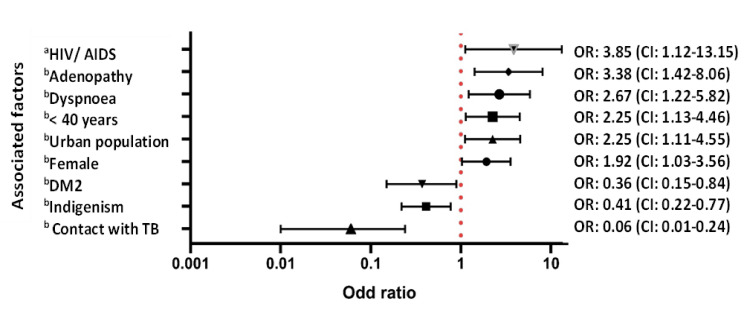
Adjusted ORs for the factors associated with EPTB+ compared to PTB+ ^a^OR adjusted for age, sex, indigenism, SES, tobacco, alcohol and drug use, type of population, BMI, and previous contact with TB ^b^OR adjusted for age, sex, indigenism, SES, tobacco, alcohol and drug use, diabetes, HIV/AIDS, type of population, BMI, drug resistance, presence of adenopathy, and dyspnea HIV/AIDS, human immunodeficiency virus/acquired immunodeficiency syndrome; T2DM, type 2 diabetes mellitus; BMI, body mass index; PTB, pulmonary tuberculosis; HIV/AIDS, human immunodeficiency virus/acquired immunodeficiency syndrome; EPTB, extrapulmonary tuberculosis; TB, tuberculosis; SES, socioeconomic level; OR, odds ratio

However, after adjustment, being female, being <40 years old, having urban residence, comorbidity with HIV/AIDS, dyspnea, and adenopathy maintained the association with EPTB+. While indigeneity, presence of diabetes, and previous contact with TB subjects decreased the probability of EPTB+ (Figure 2).

## Discussion

According to our results, subjects with EPTB+ were younger compared to those with PTB+, also, their SES was higher and they were residents of urban areas. Nodal, miliary, and breast TB were the most frequent clinical presentations. Age <40 years, female sex, urban residence, comorbidity with HIV/AIDS, dyspnea, and adenopathy were positively associated with EPTB+, whereas indigeneity, diabetes, and previous contact with TB patients decreased its likelihood.

The main clinical manifestation of EPTB+ was lymph node (20.1%), which is in line with other studies from Turkey, the United States, the United Kingdom, Nepal, and Mexico [7-9,12,14]. However, this is contradicted by reports in the Asian population, where the main site of EPTB+ was bone but in other studies in Europe, the pleura was the most affected site [15,16]. The second most frequent clinical presentation was miliary TB. Miliary TB is considered a manifestation with high mortality, and previous studies have reported that it mainly occurs among the young population, which is consistent with our observations [17]. An interesting finding was that breast TB was the third most frequent clinical presentation, which is a rare entity even in endemic areas and has had a low frequency in previous studies [7-9,12,17,18]. This may be due to the difficulty in diagnosing this type of TB, as it can mimic benign conditions such as fibroadenoma as well as malignant conditions such as carcinoma [19]. In addition, it is difficult to distinguish mammary TB from granulomatous mastitis, and from this premise, almost 60% of the cases in our study were diagnosed radiologically so it was not possible to confirm histopathologically to rule out other possible pathological entities [19].

In addition to the above, the differences observed between the frequency of the different clinical presentations of EPTB+ could be related to geographic, ethnic, environmental, genetic, and specific factors of the circulating strains of Mycobacterium tuberculosis [1,7]. For instance, several studies investigating the molecular characteristics of EPTB+ strains in China reported the presence of the Beijing genotype in 88% of bone TB cases and 80% of tuberculous meningitis cases; this genotype is associated with increased virulence as well as drug resistance and circulates mainly in Asia and Europe, which could explain the differences between the main sites of EPTB+ produced by different strains in different geographical areas [20,21].

On the other hand, BCG vaccination has been associated with a lower probability of dangerous clinical presentations such as miliary and meningeal TB in children; however, the frequency of miliary TB in our study implies that this effect is lost in adulthood, as most patients with EPTB+ had a history of BCG vaccination (p=0.022) [3]. This is in line with that reported in a systematic review and meta-analysis with 68,558 participants, which concludes that BCG vaccination only protects 18% of children under five years of age against TB in any form, reflecting the urgency of new vaccines for TB control and prevention worldwide [22].

Moreover, age <40 years was positively associated with EPTB+, which is consistent with that reported in Germany, China, and Turkey [7,23,24]. It has also been documented that in countries with a high TB burden such as Nepal and Mexico, a mean age between 25 and 32 years was associated with EPTB+ [12,14]. Furthermore, age has been associated with different sites of affection, for example, EPTB+ in bones, and the genitourinary system has been observed in people over 40 years of age, while lymphatic and breast infections in children occurring under this age range [25]. This could be because the young population has a less mature immune system, which could facilitate EPTB+ spread. For example, a study conducted in Nepal observed that after primary infection in the lungs, the probability of reactivation in an extrapulmonary site was higher among young people, while for older ages TB reactivation in the lungs was more common [12]. The reason that older adults are less prone to EPTB+ may be related to changing immune function and adaptations throughout life; however, there is no consensus on the effect of aging on the development of EPTB+ [15].

Likewise, women were more likely to present with EPTB+ compared to men, and this has been previously documented in other studies in the United States, Nepal, Turkey, and Germany [7,8,12,23]. The underlying mechanisms that may explain the sex difference are not very clear, as there are conflicting data. For example, in a large study in China (n=202,998), it was observed that EPTB+ was mainly associated with males, and in another study in Mexico, no significant differences were observed between males and females with respect to the frequency of EPTB+ [14,24]. However, some authors point out that the differences reported in various studies are related to endocrine and cellular immunity factors, iron metabolism, financial barriers, and access to medical services, as well as social stigma about the disease [25]. Others conclude that women in high TB-burden countries may be an independent factor for EPTB+; therefore, the independent effect of sex should be studied in depth in future studies [12].

Another factor that increased the probability of EPTB+ was urban origin, which coincides with another study in Malaysia but contradicts the results of another study in China [15,26]. However, a limitation in the search for an association with this variable is that the criteria for categorizing rural and urban areas are not homogeneous, which could affect the proportion of patients in each category and, therefore, the association.

In this study, comorbidity with HIV/AIDS increased the likelihood of EPTB+ and is similar to that reported in the literature, which could be explained by the developed immunosuppression and dysfunction of innate immunity [16,25,27]. Therefore, TB screening in HIV/AIDS patients is crucial for the control of EPTB+. Other pathologies that cause immunosuppression such as DM2 have been reported as a risk factor for the development of PTB+; in our study we found that having DM2 was a factor that increased the probability of PTB+ but decreased that of EPTB+, being similar to what was observed in another study in China [15,28]. However, the mechanisms to explain these discrepancies are not clear. One hypothesis could be that age distorts the true association between these variables since as mentioned, in general, patients with EPTB+ were younger so it is likely that they have not yet developed this chronic disease. In addition, it is important to consider dysglycemia since it could generate a greater susceptibility to develop active TB; for example, in a study carried out with patients from four cities in South Africa, Indonesia, Peru, and Romania, it was reported that individuals with intermediate hyperglycemia (IH) present immune dysfunctions related to interferon I, similar to individuals with diabetes; therefore, studies focused on EPTB+ dynamics in dysglycemic conditions and TB surveillance systems focused not only on established DM2 but also on IH and glycemic control are needed [29].

Although EPTB+ is less contagious due to its characteristics, it can eventually spread to the lungs and become infectious (PTB+), which represents a latent threat to public health and global TB control [10]. EPTB+ also results in a significant number of hospitalizations, longer in-hospital time, and higher costs compared to PTB+, generating a significant impact on patients' lives [30]. In addition, the clinical and symptomatological characteristics of EPTB+ can vary according to the organ or tissue affected, so both the general and specific manifestations of the disease must be considered. This is one of the reasons why the diagnosis of it is complex, which can delay it and increase the probability of developing antibiotic-resistant strains; therefore, the correct identification of risk factors for EPTB+ that could facilitate early diagnosis is of vital importance [25].

Our study has some limitations, for instance, the nature of the retrospective design based on a secondary source could have given rise to information bias in addition to the fact that it was not possible to evaluate variables of interest such as glycemic control, vitamin D deficiency, or the use of immunosuppressive drugs. Furthermore, the sample size can be considered small; however, the 1:4 ratio of cases and controls improves statistical efficiency. In contrast, the similarity of our results with previous research lends support to our findings; in addition, to our knowledge, this is the first study in the region and one of the few in the country to address factors associated with EPTB+, which provides a basis for future research.

## Conclusions

Despite its lower prevalence compared to PTB, EPTB can complicate the patient's prognosis. Due to the atypical clinical symptoms, difficulty in obtaining specimens, and low positive rate of etiology in EPTB, the diagnosis of EPTB is very difficult and easy to misdiagnose. Knowing the factors associated with this disease could contribute to the development of screening tools for the identification and timely diagnosis of probable sick patients.

In summary, this study made it possible to describe the risk factors associated with EPTB+ in patients from the Isthmus of Tehuantepec region, Oaxaca, Mexico, which could be used as a basis for screening EPTB+, thus improving the preventive and diagnostic capacity of local health services, taking as a starting point women under 40 years of age and patients with HIV/AIDS in urban areas as well as the presence of adenopathy and dyspnea as clinical characteristics of the disease.
